# Hydration-Controlled Coupling of Water Polarization and Proton Transport in Nafion Under External Electric Fields

**DOI:** 10.3390/polym18192330

**Published:** 2026-09-24

**Authors:** Daniel P. Luis-Jiménez, Luis A. Martínez-Santiago, Jonathan Toledo-Toledo, Jorge López-Lemus, Alfredo González-Calderón, Damazo N. Jiménez-Antonio

**Affiliations:** 1 SECIHTI–Instituto Tecnológico del Istmo, Tecnológico Nacional de México, Juchitán de Zaragoza 70000, Oaxaca, Mexico; 2 Instituto Tecnológico de Oaxaca, Tecnológico Nacional de México, Oaxaca de Juárez 68030, Oaxaca, Mexico; luis.martinez@itoaxaca.edu.mx; 3 Instituto Tecnológico del Valle de Etla, Tecnológico Nacional de México, Santiago Suchilquitongo 68230, Oaxaca, Mexico; jonathan.toledo@infotec.mx; 4 INFOTEC Centro de Investigación e Innovación en Tecnologías de la Información y Comunicación, Mexico City 14050, Mexico; 5 Facultad de Ciencias, Universidad Autónoma del Estado de México, Toluca 50200, Estado de México, Mexico; jllemus@uaemex.mx; 6 SECIHTI–Department of Geomatic and Hydraulic Engineering, University of Guanajuato, Guanajuato 36000, Guanajuato, Mexico; jagonzalezca@secihti.mx; 7 Instituto Tecnológico del Istmo, Tecnológico Nacional de México, Juchitán de Zaragoza 70000, Oaxaca, Mexico

**Keywords:** Nafion, proton exchange membrane, molecular dynamics, external electric field, dielectric constant, hydronium transport, hydration, electrostatic screening

## Abstract

We use molecular dynamics simulations at a fixed cell volume (canonical, NVT, and ensemble) to study hydrated Nafion at hydration levels λ=3,9,15 under external electric fields. At zero field, the static dielectric constant of the aqueous subsystem, obtained from water dipole fluctuations, grows with hydration, from ϵ≈2.9 at λ=3 to ϵ≈17.2 at λ=15; because the single-molecule dipole is unchanged, this enhancement is cooperative, reflecting dipolar correlations that build up as the aqueous domains percolate. Under an applied field, the fluctuation formula no longer defines an equilibrium dielectric constant, so we characterize the response through the polarization itself: the collective water dipole orients along the field already at low fields, while its magnitude $\langle M_{z}\rangle$ saturates gradually, on a field scale that grows with hydration. This polarization build-up is accompanied by a reorganization of the first H_3_O^+^–SO_3_(H) coordination shell and by a crossover from diffusive to drift-dominated proton transport. These signatures shift together to higher fields as hydration increases, consistent with a common controlling variable: electrostatic screening by the added water. Because volume is held fixed across λ, the results characterize this coupling at controlled density rather than at each hydration’s equilibrium swelling volume.

## 1. Introduction

Proton exchange membrane fuel cells (PEMFCs) are among the most promising energy conversion devices for sustainable power generation, relying on the efficiency and durability of proton-conducting membranes such as Nafion. Nafion, a perfluorosulfonic acid (PFSA) polymer, combines a hydrophobic polytetrafluoroethylene (PTFE) backbone with perfluoroether side chains terminated by sulfonic acid groups, forming a complex nanophase-separated morphology that governs water uptake and ion transport [1,2,3,4]. Understanding the molecular-scale structure and dynamics of Nafion is therefore essential for the design of improved membranes with enhanced proton conductivity and mechanical stability, an area that remains under intense development [5].

This nanophase-separated picture was established well before atomistic simulation became available, through classical scattering and transport studies of the polymer. Gierke and coworkers proposed, on the basis of small- and wide-angle X-ray scattering, a cluster–network model in which sulfonic acid groups aggregate into roughly spherical ionic clusters a few nanometers across, interconnected by narrow channels that percolate at sufficient hydration [6,7], a picture later reviewed comprehensively by Mauritz and Moore [8]. Subsequent small-angle scattering studies refined this view, favoring elongated, rod-like polymeric aggregates over isolated spherical clusters [9], while solid-state NMR measurements suggested instead that the water-filled domains form parallel, roughly cylindrical nanochannels [10]. These classical, macroscopic structural pictures—cluster–network, fibrillar, and channel-like—remain the experimental reference against which atomistic models of Nafion, including the present one, are ultimately judged.

Atomistic and coarse-grained molecular dynamics (MDs) simulations have played a crucial role in elucidating the relationship between hydration, morphology, and transport phenomena in Nafion. Early simulation studies revealed the existence of continuous water domains within the hydrophilic regions and provided insight into the formation of interconnected channels facilitating proton transport [1,11]. Subsequent works introduced more refined models of hydrated Nafion, demonstrating how sulfonic acid aggregation and water clustering drive nanophase segregation [2,3]. Jinnouchi et al. [12] further established that the mobility of hydronium ions is strongly coupled to the dynamic reorganization of the polymer–water interface, while later simulations clarified oxygen permeation and gas solubility in the membrane under operational conditions [13]. Atomistic simulation remains an active tool in this area: recent studies have resolved proton transport across the interfaces of the nanophase-separated domains in short-side-chain ionomers such as Aquivion [14] and characterized transport in Nafion-based composite membranes [15].

More recent studies have focused on the morphological response of Nafion thin films at electrode interfaces. Kang et al. [16] used molecular dynamics to resolve how Nafion nanostructure and side-chain orientation reorganize at the platinum/carbon catalyst surface, while Cheng et al. [17] showed, also from molecular dynamics, that the platinum nanoparticle size itself shapes the resulting catalyst-layer microstructure. These results reveal the multiscale complexity of Nafion morphology and point to the need for accurate atomic-level models that capture both electrostatic interactions and dielectric polarization effects. More recently, molecular dynamics has also been used to probe how external electric fields, together with temperature and hydration, affect proton transport in ordered and amorphous proton exchange membranes [18]; the field response of these materials is attracting growing attention. These studies, however, report the field dependence of transport largely on its own, without also tracking the dielectric and structural response of the membrane under the same field and hydration conditions; whether these responses share a common, hydration-set field scale, rather than shifting independently, has not been established.

In this work, we extend previous atomistic modeling efforts by performing density functional theory (DFT) calculations to obtain Hirshfeld-derived atomic charges for Nafion monomer units, which were subsequently used in classical molecular dynamics simulations with GROMACS. Rather than treating the dielectric, structural, and transport properties of hydrated Nafion as separate observables, we focus on how they are coupled under an external electric field. We show that the equilibrium dielectric response of the aqueous phase, the build-up and saturation of the field-induced water polarization, the reorganization of the hydronium coordination shell, and the onset of directed hydronium transport shift together rather than independently, and that this concerted shift is consistent with a single controlling variable: the degree of electrostatic screening set by the water content. More water means stronger screening of the attraction between the mobile ions and the sulfonate network, and the characteristic field of each of these responses moves upward accordingly. By scanning hydration levels (λ) and external fields, we thus map the coupling between water polarization and proton transport that governs the field response of perfluorinated ionomer membranes.

## 2. Molecular Models and Method

The Nafion monomer employed in the present simulations consists of a perfluorinated PTFE-like backbone composed of ${\mathrm{CF}}_{x}$ units, bearing four perfluoroether side chains terminated by sulfonic acid groups (−SO_3_H), as shown in Figure 1. The detailed chemical structure is presented in Figure 2. In this molecular model, four sulfonated side chains are covalently attached to the fluorocarbon backbone at regular intervals. The resulting repeat unit has a molecular weight of 3215 g ${\mathrm{mol}}^{-1}$ and an equivalent weight of EW = 804 g ${\mathrm{eq}}^{-1}$. This value is lower than that of standard Nafion 117 (EW ≈ 1100 g ${\mathrm{eq}}^{-1}$). Importantly, our model retains the standard Nafion long side chain (Figure 2); the reduced EW arises from a higher density of these side chains along the backbone, not from side-chain shortening. It thus represents the high-sulfonation-density end of the long-side-chain Nafion family, and although this EW coincides with the range of short-side-chain ionomers such as Aquivion/Hyflon (EW ≈ 700–870 g ${\mathrm{eq}}^{-1}$) [19], the two differ in side-chain chemistry. The higher density of sulfonic groups increases the concentration of exchange sites per unit mass, which is expected to promote water clustering and the percolation of ionic domains at a given hydration level λ. We do not vary the equivalent weight in this work; accordingly, the absolute field scales reported below may shift for membranes of higher EW, while the coupling mechanism we describe is expected to be general.

The bulk Nafion system was modeled by placing 36 Nafion monomers together with 10 hydronium (H_3_O^+^) and 10 hydroxide (OH^−^) ions, as well as a number of water molecules (H_2_O) set by the hydration level λ, defined as the number of water molecules per sulfonic acid group. With four sulfonic groups per monomer and 36 monomers, the system contains 144 sulfonic acid groups in total. In the present topology, these groups are modeled in their protonated −SO_3_H form so that each monomer is electrically neutral, and the corresponding number of water molecules is 144λ, so that a higher λ corresponds to a larger water content.

The ionic composition itself deserves a remark. The mobile carriers consist of a small, fixed population of 10 H_3_O^+^ and 10 OH^−^ ions so that the system is charge-neutral by construction. This differs from the fully dissociated limit expected for a perfluorosulfonic superacid at these hydration levels, in which each sulfonic group would donate its proton to the aqueous phase, yielding on the order of order 144 H_3_O^+^ and 144 SO_3_^−^ per box. What our setup probes, therefore, is the field response of a dilute carrier population within a predominantly protonated matrix, not the transport of a fully dissociated membrane. The diffusion coefficients and drift thresholds reported below should be read in that light: they characterize the field-driven mobility of the explicit carriers and the reorganization of the H_3_O^+^–SO_3_(H) coordination and provide a lower bound on the proton transport of the fully hydrated, fully dissociated membrane.

All systems were contained in an orthorhombic simulation box of fixed dimensions $4.80\times 4.50\times 6.80{\mathrm{nm}}^{3}$, common to every hydration level. Periodic boundary conditions were applied in all three spatial directions to mimic bulk behavior, as illustrated in Figure 3.

The bonded and Lennard–Jones parameters of the Nafion monomer were taken from the GROMOS 54A7 united-atom force field [20], the initial topology being generated with the Automated Topology Builder (ATB) [21,22]. Non-bonded interactions follow the GROMOS geometric combination rules. The atomic charges provided by the ATB were then replaced by charges derived from a Hirshfeld population analysis [23], following the strategy described in Refs. [24,25,26]. The electronic structure calculations were performed using Density Functional Theory (DFT) with the ORCA 5.0.4 package [27], employing the B3LYP functional and the def2-SVP basis set. Geometry optimizations were followed by single-point energy calculations, and the Hirshfeld method was applied to extract the partial atomic charges. These charges were uniformly scaled by a factor of 1.2 to reproduce the reference molecular dipole moment [24], and the scaled values were assigned to the Nafion topology.

The molecular dynamics (MDs) simulations were conducted with the GROMACS 2024.5 software [28]. Systems consisting of hydrated Nafion were constructed using different hydration levels, characterized by the hydration parameter λ (number of water molecules per sulfonic acid group). Values of λ = 3, 9 and 15 were studied to represent different levels of membrane hydration. Water was represented by the rigid four-site TIP4P/Ice model [29], while the hydronium (H_3_O^+^) and hydroxide (OH^−^) ions were represented by rigid, non-polarizable models carrying formal charges of +1 and −1, respectively, with Lennard–Jones interactions assigned only to the oxygen atom. Following the approach of Elliott et al. [11], who built their hydronium fragment directly from the water molecule to preserve internal consistency with the solvent, the ionic oxygen Lennard–Jones parameters were taken from the SPC/E water model [30], and the ion geometry was inherited from a standard water model. This entails a slight mismatch between the van der Waals description of the ions and that of the TIP4P/Ice solvent; because the ions form a dilute species and the dielectric analysis is restricted to the water subsystem, we expect this mismatch to have a limited effect on the reported trends, but state it explicitly as a limitation. We note that TIP4P/Ice was parametrized to reproduce the phase diagram of ice rather than the dielectric or dynamical properties of ambient liquid water; owing to its four-site charge distribution, it yields, at 300 K, a lower static dielectric constant and a slower self-diffusion than real water. The dielectric and transport quantities reported below should therefore be read against the intrinsic bulk behavior of this model, not against experimental water directly. After energy minimization and a 2.5 ns equilibration ($5\times 10^{6}$ steps), production runs were carried out in the NVT ensemble at 300 K, with the temperature maintained by a Nosé–Hoover thermostat ($\tau_{T}=0.05$ ps). The equations of motion were integrated with a leap-frog algorithm and a time step of 0.5 fs, and each production trajectory spanned 10 ns ($2\times 10^{7}$ steps). A Verlet cutoff scheme was used with a common cutoff of 1.0 nm for the Lennard–Jones and real-space electrostatic interactions; long-range electrostatics were treated with the Particle Mesh Ewald (PME) method [31], and a long-range dispersion correction was applied to both energy and pressure. All bonds involving hydrogen were constrained with the LINCS (LINear Constraint Solver) algorithm, and the water and ion geometries were kept rigid. The main simulation parameters are summarized in Table 1.

An external electric field was applied along the *z* direction. In the presence of the field, the total potential energy of the system is expressed as

(1) $$U_{\mathrm{total}}=\sum_{i<j}U_{ij}-E\sum_{i}q_{i}z_{i},$$

where *E* is the magnitude of the external electric field, $q_{i}$ is the partial charge of particle *i*, and $z_{i}$ is its Cartesian coordinate along the *z* direction. The first sum runs over all pairs of interaction sites i,j, and $U_{ij}$ represents the intermolecular interaction energy between sites *i* and *j*, including electrostatic and Lennard–Jones contributions. This external-field scheme is the same one we have employed in previous molecular dynamics studies of electric-field effects on clathrate hydrates [32,33].

The electric field strength was varied between 0 and 4.0 V ${\mathrm{nm}}^{-1}$, with the specific values chosen for each hydration level listed in Table 2. The field magnitudes explored here (up to 4.0 V ${\mathrm{nm}}^{-1}$) are several orders of magnitude larger than the average macroscopic field across an operating PEMFC membrane ($∼10^{-4}$–$10^{-2}$ V ${\mathrm{nm}}^{-1}$). Such elevated fields are a standard device in non-equilibrium molecular dynamics of membranes and electrolytes [18,32,33]: they are required to drive a measurable ionic response within the accessible simulation time, and they probe the local electrostatic scale at which the sulfonate–ion coupling operates. The drift thresholds reported below should therefore be read as a probe of the intrinsic coupling between polarization and transport, not as literal operating conditions; the physically meaningful result is the systematic shift of that coupling with hydration, not the absolute field values. All simulations were carried out in the canonical (NVT) ensemble at a constant temperature of 300 K and fixed volume. A single simulation cell of fixed dimensions was used for all hydration levels and field strengths, and no constant-pressure (NPT) equilibration was performed. This choice is deliberate. Under the strong static external fields applied here, the field continuously does work on the net-mobile ionic species and drives the system into a field-driven steady state; barostat-based pressure control, which presupposes mechanical equilibrium and an unbiased virial, is therefore not well defined under the field, and a fixed-volume ensemble is the controlled and reproducible setting for a systematic scan over λ and *E*. Using an identical cell for every system further removes the volume as a confounding variable: differences in the computed observables reflect hydration and field, not concurrent changes in cell size, and the zero-field dielectric reference and the field response are evaluated in the same cell. A consequence of this protocol is that, since water is added to a cell of fixed size, the mass density increases with hydration, from 1.40 to 1.58 and 1.75 g ${\mathrm{cm}}^{-3}$ for λ=3, 9 and 15. We therefore treat the systems as a controlled-volume series rather than as reproductions of the experimental swelling isotherm; because absolute transport coefficients depend on density, the cross-λ comparison below should be read as an analysis of the coupling and of the trends at fixed volume, not as a prediction of absolute mobilities at each hydration’s equilibrium density. For each λ value, the total dipole moment, its longitudinal component, and the Kirkwood factor were extracted from the trajectories. The static dielectric constant was obtained from the Kirkwood–Fröhlich dipole fluctuation formula implemented in GROMACS under tin-foil (conducting) boundary conditions,

(2) $$\epsilon =1+\frac{\langle M^{2}\rangle -{\langle M\rangle }^{2}}{3\;\varepsilon_{0}Vk_{B}T},$$

where M is the total dipole moment of the aqueous subsystem, $\varepsilon_{0}$ is the vacuum permittivity, *V* is the (fixed) simulation-cell volume, $k_{B}$ is the Boltzmann constant, and *T* is the temperature. We restrict the fluctuation formula to the water molecules because the mobile ionic species (H_3_O^+^ and OH^−^) carry a net charge: their displacement contributes to the ionic conductivity rather than to the orientational polarization, and including them would conflate the two responses and make M ill-defined under periodic boundary conditions. This expression is a linear-response, equilibrium relation: it is physically meaningful only in the absence of an external field, where the spontaneous dipole fluctuations sampled by the simulation define the zero-field susceptibility. Under a finite field, the dipoles align and saturate, the fluctuations are suppressed, and the formula no longer yields a dielectric constant. We therefore report ϵ only at E=0; the field-dependent polarization response is characterized instead by $\langle M_{z}\rangle$ and the alignment fraction $|M_{z}\left|/|M\right|$, with the finite-volume Kirkwood factor $G_{k}$ used only as an apparent indicator of dipolar correlation. These analyses enabled the evaluation of the structural, polarization, and transport response of Nafion under different hydration states and external field conditions.

Statistical uncertainties on the zero-field dielectric constant were estimated by block averaging: the 8 ns of production sampled for the dipole (the first 2 ns being discarded as equilibration) was divided into four 2 ns blocks, and the reported uncertainty is the standard deviation across blocks. Our full-trajectory ϵ reproduces the built-in GROMACS estimate to within numerical precision at all three hydration levels, confirming the implementation. The block-to-block scatter is smallest at low hydration (λ=3: ϵ=2.9±0.3) and largest at high hydration (λ=15: ϵ=17.2±2.9). This trend follows the collective nature of the dielectric response: the autocorrelation time of the aqueous dipole grows from ≈0.19 ns at λ=3,9 to ≈0.37 ns at λ=15, so that each fixed-length block contains correspondingly fewer independent samples of the slowly relaxing total dipole M. The high-ϵ, strongly correlated regime is therefore intrinsically the hardest to converge, and the same collective correlations that enhance ϵ also govern its slower convergence.

This slower convergence is specific to the zero-field dielectric constant, which is obtained from the dipole-fluctuation formula and is therefore directly sensitive to the correlation time of the total dipole. The drift threshold and the H_3_O^+^–SO_3_(H) coordination numbers reported in Section 3.2 and Section 3.4 are not obtained from a fluctuation formula: *D* is a direct slope of the block-averaged mean squared displacement, and the coordination numbers are direct block averages of the RDF integral, neither of which amplifies the slow tail of the dipole autocorrelation function the way $\langle M^{2}\rangle -{\langle M\rangle }^{2}$ does. Consistent with this, the block-to-block uncertainty on the λ=15 coordination numbers is ≲15% of the mean (Section 3.4), well below the ≈17% scatter of ϵ at the same λ, even though the latter is built from second moments of the same slowly relaxing dipole. More importantly, the drift threshold is identified from a qualitative, large-amplitude feature, an increase of the apparent *D* by two to three orders of magnitude between consecutive field points, that is far larger than any plausible statistical fluctuation from finite sampling; reproducing such a shift by chance would require an implausibly large sampling error. We also note that, at λ=15, the drift threshold in D(E), the maximum of the S–H_3_O^+^ RDF peak height $g_{1}$, and the saturation of $\langle M_{z}\rangle$ all occur over the same narrow field window (Section 3.2), even though these three quantities are computed from independent trajectories and independent block-averaging procedures; such cross-observable agreement would be unlikely if the λ=15 results were dominated by sampling noise rather than by a genuine field-driven crossover. We conclude that the longer dipole relaxation time at λ=15 inflates the uncertainty of the equilibrium ϵ specifically, without undermining the drift-threshold or coordination-shell conclusions, which rest on independently sampled, non-fluctuation-based observables.

## 3. Results and Discussion

The high sulfonate density has two direct consequences for ion transport. First, even in the absence of an applied field, the H_3_O^+^ ions at low hydration are already embedded in a dense network of neighboring sulfonic groups, which templates the vehicular motion of the intact H_3_O^+^ ions through the sulfonic-group array. This is reflected in the zero-field diffusion coefficients, which decrease monotonically with hydration: D(E=0)≈0.023, 0.006, and $0.00008\times 10^{-5}{\mathrm{cm}}^{2}s^{-1}$ for λ=3, 9, and 15, respectively. The progressive dilution of the sulfonate network by water thus reduces the intrinsic, field-free proton mobility as hydration increases. We emphasize that this trend is a feature of the controlled-volume protocol (Section 2): because the cell size is held fixed, increasing λ raises the mass density (from 1.40 to 1.75 g ${\mathrm{cm}}^{-3}$), so the added water crowds the fixed cell and reduces the free volume available for ion motion. The decreasing zero-field mobility therefore reflects this increasing confinement; it does not contradict the enhanced conductivity observed in constant-pressure, freely swelling membranes.

Second, the water content controls the minimum field required to drive H_3_O^+^ out of the diffusive regime into field-driven drift. As additional water molecules are incorporated, they progressively screen the electrostatic interaction between the sulfonate groups and the mobile ions, so that a stronger external field is needed to overcome the structural constraints of the hydrated matrix. This screening is quantified by the systematic shift of the drift threshold: E≈0.7, 1.4, and 3.7 V ${\mathrm{nm}}^{-1}$ for λ=3, 9, and 15, respectively (see Section 3.2). Below each threshold, however, the field does enhance diffusive transport. The relative enhancement (the factor by which *D* increases from zero field to the diffusive maximum) grows with hydration, reflecting the larger structural reorganization that the field induces in more water-rich environments.

These observations are consistent with the classical cluster–channel model proposed by Hsu and Gierke [7] for Nafion membranes, in which sulfonic acid groups aggregate into hydrophilic ionic clusters interconnected by narrow channels. In the present model, the high sulfonate density (EW = 804 g ${\mathrm{eq}}^{-1}$) promotes well-defined ionic domains even at moderate hydration levels. The applied field enhances the longitudinal connectivity of these domains by partially aligning the hydrated ionic regions, but the field strength required to achieve this alignment is set by the degree of electrostatic screening, which increases monotonically with λ. The quantitative thresholds reported here thus provide a direct measure of how membrane hydration controls the field-driven onset of directed hydronium transport.

### 3.1. Zero-Field Dielectric Response and Field-Induced Dipolar Alignment

At zero field, where the fluctuation formula defines a genuine equilibrium dielectric constant, the aqueous-subsystem ϵ grows monotonically with hydration, from ϵ≈2.9±0.3 at λ=3 to ϵ≈6.0±0.3 at λ=9 and ϵ≈17.2±2.9 at λ=15 (Figure 4). This growth is driven by collective dipolar correlations: the static Kirkwood factor $G_{k}$ exceeds unity and increases with hydration, whereas the water molecular dipole is fixed by the (rigid) water model. The dielectric enhancement with hydration therefore reflects the increasing mutual alignment of the water dipoles as the aqueous domains percolate, not a change in the single-molecule dipole.

These values should be read relative to the bulk dielectric constant of the water model itself. Owing to its four-site charge distribution, TIP4P/Ice yields a static dielectric constant below that of experimental water at 300 K, so the growth from ϵ≈2.9 at λ=3 to ≈17.2 at λ=15 reflects the progressive build-up of the aqueous dielectric response toward the model’s own bulk value as the hydrophilic domains percolate; it is not an approach to the experimental bulk permittivity.

Under a finite field, the dipole-fluctuation formula no longer defines a dielectric constant: the field aligns the dipoles and suppresses the very fluctuations on which the linear-response relation rests, so the quantity it returns loses its physical meaning as ϵ. We therefore characterize the field response directly through the polarization of the aqueous subsystem. The longitudinal water dipole $\langle M_{z}\rangle$ rises steeply at low fields and then saturates as the dipoles become fully aligned (Figure 5); for λ=15, for instance, $\langle M_{z}\rangle$ grows from essentially zero at E=0 to ≈3000 D already at E=1 V ${\mathrm{nm}}^{-1}$, and then approaches its saturation value of ≈4500 D only gradually, with increments of a few percent per field step beyond E≈3 V ${\mathrm{nm}}^{-1}$. Both the saturated dipole and the field needed to approach it grow with hydration: the λ=3 and λ=9 curves approach saturation by E≈1.5 and 2 V ${\mathrm{nm}}^{-1}$, respectively, whereas for λ=15, the approach extends to E≈3–4 V ${\mathrm{nm}}^{-1}$. The response is strongly anisotropic, with the longitudinal component dominating over the transverse ones for all λ and *E*, and the two observables separate the orientation of the collective dipole from its magnitude. The alignment fraction $|M_{z}\left|/|M\right|$ jumps from its near-isotropic zero-field value to ≳0.99 already at E=0.5 V ${\mathrm{nm}}^{-1}$ for λ=9 and 15, and above ≈0.98 by E≈0.7 V ${\mathrm{nm}}^{-1}$ for λ=3 (Figure 6): the direction of the collective water dipole locks onto the field essentially immediately, at fields well below those at which its magnitude saturates. The continued growth of $\langle M_{z}\rangle$ at higher fields therefore reflects the progressive alignment of the individual molecular dipoles within an already field-oriented collective moment, and it is this magnitude saturation, not the orientational locking, that carries the hydration-dependent field scale, consistent with field-induced ordering amplified by the percolation of the aqueous domains.

This field-driven alignment does not occur in isolation: it is coupled to the structural and transport reorganization characterized in the following sections, all sharing a common field scale, which is again set by hydration through electrostatic screening: the more water is present, the stronger the field required to align the ionic network and to drive the carriers out of the diffusive regime. Concretely, for λ=3, the diffusion coefficient *D* reaches its diffusive-regime maximum at E≈0.6 V ${\mathrm{nm}}^{-1}$, the drift onset occurs at E≈0.7 V ${\mathrm{nm}}^{-1}$, and the S–H_3_O^+^ first-peak height $g_{1}$ peaks at E≈1.0 V ${\mathrm{nm}}^{-1}$, all within the same narrow window; for λ=9, the diffusive maximum of *D* (E≈1.0 V ${\mathrm{nm}}^{-1}$) and the field-induced recovery maximum of $g_{1}$ (E≈1.5 V ${\mathrm{nm}}^{-1}$) likewise cluster around the drift onset at E≈1.4–1.6 V ${\mathrm{nm}}^{-1}$, and for λ=15, the same sequence is displaced to E≈3.7 V ${\mathrm{nm}}^{-1}$. That the maxima of cooperative diffusion and of first-shell H_3_O^+^–SO_3_(H) occupancy track each other as λ varies points to a common origin for the polarization, structural, and transport responses: a screening-controlled build-up of correlated ionic ordering.

### 3.2. Hydronium Transport Coupled to the Polarization Response

The polarization response identified above is directly coupled to ion transport. To make this coupling explicit, we extracted the diffusion coefficients *D* of hydronium ions from the linear regime of the mean squared displacement (MSD) curves, fitting the time interval between 2000 and 6000 ps. This window excludes the initial ballistic regime and minimizes the nonlinear effects that emerge at long times under strong external fields. Because a uniform field exerts a net force on the charged carriers, the long-time motion ceases to be purely diffusive once the field is strong enough; we therefore restrict the quantitative analysis to the diffusive regime and treat the large-field data separately as a drift threshold (see below). The resulting D(E) curves for λ=3, 9 and 15 are shown in Figure 7, and the corresponding values are listed in Table 2.

Within the diffusive regime, D(E) displays a nonmonotonic, hydration-dependent behavior that parallels the build-up of the water polarization described above. For λ=3, *D* first decreases weakly as the field rises from zero ($0.023\to 0.018\times 10^{-5}\;{\mathrm{cm}}^{2}\;s^{-1}$ between E=0 and $0.3\;V\;{\mathrm{nm}}^{-1}$), then rises sharply to a local maximum near $E\approx 0.6\;V\;{\mathrm{nm}}^{-1}$ ($1.74\times 10^{-5}\;{\mathrm{cm}}^{2}\;s^{-1}$). For λ=9 the same feature appears but shifted to higher field: *D* peaks at $E\approx 1.0\;V\;{\mathrm{nm}}^{-1}$ ($0.66\times 10^{-5}\;{\mathrm{cm}}^{2}\;s^{-1}$) and is then suppressed at E=1.2–$1.3\;V\;{\mathrm{nm}}^{-1}$ (0.33 and $0.27\times 10^{-5}\;{\mathrm{cm}}^{2}\;s^{-1}$). This local-maximum-then-suppression structure reflects the same cooperative ordering that underlies the growth of the water polarization, and it progressively weakens with hydration: for λ=15, it survives only as a shallow plateau near E=1.5–$2.0\;V\;{\mathrm{nm}}^{-1}$ (0.023 and $0.022\times 10^{-5}\;{\mathrm{cm}}^{2}\;s^{-1}$), where the variation falls within the scatter of the fits and should be regarded as indicative rather than conclusive.

The most robust trend is the systematic shift of the field scale with hydration. The onset of field-driven drift, identified by the abrupt rise of the apparent *D* by several orders of magnitude, moves to progressively higher fields as λ increases: $E\approx 0.7\;V\;{\mathrm{nm}}^{-1}$ for λ=3, E≈1.4–$1.6\;V\;{\mathrm{nm}}^{-1}$ for λ=9, and E≈3.7–$4.0\;V\;{\mathrm{nm}}^{-1}$ for λ=15. This monotonic displacement is a direct manifestation of the electrostatic screening discussed above: the additional water progressively screens the interaction between the sulfonate groups and the mobile ions so that a stronger field is required to overcome the structural constraints of the hydrated matrix and drive the carriers into the drift regime. The same screening that sets the field scale of the water polarization thus also displaces the transport threshold: the two responses move together because they share their origin.

Beyond these onset fields, the apparent diffusion coefficients increase by several orders of magnitude and no longer describe equilibrium diffusion: the motion is dominated by field-driven drift rather than thermal diffusion. These large-field values are reported in Table 2 for completeness, marked with a dagger, but are excluded from the quantitative analysis above because they do not represent equilibrium transport properties.

### 3.3. The Missing Grotthuss Contribution

A limitation intrinsic to the present approach should be stated explicitly and discussed in some detail, since it bears directly on how the D(E) values and drift thresholds above should be interpreted. The force field used here is classical and non-reactive: proton transport is captured only through the vehicular motion of intact H_3_O^+^ ions, while the structural (Grotthuss) mechanism, in which the excess proton is relayed between neighboring water molecules through a sequence of hydrogen-bond reorganizations and covalent-bond switches, is entirely absent from the model. As already emphasized by Elliott et al. [11], for a similarly non-reactive Nafion model, neglecting this charge-transfer channel means that the diffusion coefficients reported here are a lower bound on the true proton mobility.

The size of this omission is not expected to be uniform across our hydration series. Reactive and experimental studies of proton transport in Nafion and related aqueous systems [34] show that the Grotthuss contribution to the total proton diffusivity is small when the aqueous domains are still sparse and disconnected and grows substantially, becoming comparable to or larger than the vehicular contribution, once the domains percolate at higher hydration. Our lowest hydration level (λ=3) corresponds to a weakly connected aqueous network (Section 2), whereas λ=9 and 15 correspond to a well-developed one; on this basis, the vehicular-only *D* values computed here likely underestimate the true proton mobility more severely at λ=9 and 15 than at λ=3. The strong decrease in D(E=0) with λ that we report in Section 3.2 should therefore be read as a lower bound whose degree of underestimation itself grows with hydration, rather than as a hydration-independent offset from the true mobility.

The consequences for the field-dependent results are more contained than for the absolute mobility. The drift threshold is identified from a qualitative, large-amplitude feature, an abrupt jump of *D* by several orders of magnitude, rather than from its absolute value, and this feature marks the field at which H_3_O^+^ escapes the electrostatic trapping of the sulfonate network, a rearrangement already captured by vehicular motion. Likewise, the polarization signature ($\langle M_{z}\rangle$, Section 2) and the H_3_O^+^–SO_3_(H) coordination-shell signature (Section 3.4) that we couple to this threshold are properties of the water dipole and of a pair correlation function, neither of which requires charge transfer to be sampled correctly. We therefore expect the reported coupling among the polarization, structural, and transport signatures, and its systematic shift with hydration, to be qualitatively robust to the absence of Grotthuss transport. The absolute field and mobility values, by contrast, would likely shift: a reactive treatment, such as the multistate empirical valence bond (MS-EVB) method [35] or ReaxFF [36], would open an additional, field-sensitive relay channel for the excess proton and would be required to determine whether it moves the drift thresholds reported here to lower fields, and by how much, particularly at λ=9 and 15.

### 3.4. Structural Signature in the First Coordination Shell

To connect the macroscopic transport behavior described above with microscopic structural changes, we computed radial distribution functions (RDFs) g(r) for three atom pairs: sulfur–sulfur (S–S), sulfur–hydronium (S–H_3_O^+^), and sulfur–water (S–W) for λ=3, 9, and 15 at the same electric fields studied in the MSD analysis. The resulting distributions are shown in Figure 8.

The S–S peak at r≈0.41 nm, which corresponds to the nearest-neighbor sulfonate distance, and the S–W peak at r≈0.38 nm remain at the same position across all fields and hydration levels, and their heights show no systematic field dependence below the drift onset: the S–S height fluctuates by ∼15–20% without a clear trend, and the S–W height is essentially constant, decreasing modestly (≈10–20%) only at the largest, drift-regime fields (Figure 8 and Figure 9). Neither the polymer backbone nor the primary hydration shell of the sulfonate groups therefore undergoes a significant field-induced structural reorganization below the drift onset.

In sharp contrast, the S–H_3_O^+^ distribution exhibits a clear and systematic response. Figure 9 shows the height $g_{1}$ of the first peak of each distribution as a function of *E* for the three hydration levels. For all λ, $g_{1}$ follows a nonmonotonic pattern that directly mirrors the ion-transport behavior: it rises toward a maximum located near the drift onset and falls once the drift regime is fully developed. For λ=3, $g_{1}$ rises from 0.75 at E=0 to 1.08 at E=1.0 V ${\mathrm{nm}}^{-1}$ and drops to 0.63 at E=1.5 V ${\mathrm{nm}}^{-1}$ (post-threshold). For λ=9, $g_{1}$ dips from 1.32 at E=0 to 0.81 at E=0.5 V ${\mathrm{nm}}^{-1}$, recovers to ≈1.1–1.2 around the drift onset (peaking at 1.21 at E=1.5 V ${\mathrm{nm}}^{-1}$), and then falls to 0.73 at E=2.0 V ${\mathrm{nm}}^{-1}$, beyond the onset at E≈1.4–1.6 V ${\mathrm{nm}}^{-1}$ where *D* has already jumped by two orders of magnitude. For λ=15, $g_{1}$ reaches its maximum value of 1.31 at E=3.0 V ${\mathrm{nm}}^{-1}$ and drops to 1.01 at E=4.0 V ${\mathrm{nm}}^{-1}$, coinciding with the abrupt rise of *D* from 0.79 to $250\times 10^{-5}{\mathrm{cm}}^{2}s^{-1}$.

This behavior identifies a two-stage structural sequence. Below the threshold, the field progressively draws H_3_O^+^ ions into closer coordination with the SO_3_(H) groups, increasing the occupancy of the first coordination shell and building a field-aligned ionic network that enhances cooperative susceptibility. Once the threshold is crossed, the ions are no longer trapped at a single sulfonate site but transition to a mobile regime in which they migrate continuously between sites; the resulting reduction in residence time at any fixed site is reflected in the drop of $g_{1}$. The drift threshold thus marks the crossover from *localized coordination* to *delocalized transport*, and the structural signature in g(r) confirms that this crossover is the common microscopic origin that couples the transport threshold in D(E) to the saturation of the field-induced dipolar alignment. The systematic shift of this threshold to higher fields with increasing λ is again a direct consequence of the electrostatic screening discussed in the preceding sections.

To complement the peak-height analysis with an integral measure, we computed running coordination numbers by integrating each RDF to a fixed cutoff $r_{c}$ taken, for every hydration level, from the position of the zero-field first minimum, so that the field dependence reflects changes in shell occupancy rather than in the integration boundary ($r_{c}\approx 1.0$ nm for λ=9 and 0.79 nm for λ=15). Uncertainties were obtained by block averaging over four 2 ns blocks, as for the dielectric analysis. Because the S–H_3_O^+^ RDF is referenced to the hydronium ions, this coordination number counts the sulfonic groups surrounding each hydronium. At λ=3, the S–H_3_O^+^ correlation remains weak ($g_{1}$ stays at ≲1.1, and the distribution lacks a clear first minimum), so no well-defined first coordination shell exists at low hydration, and the field only begins to build incipient coordination; the coordination analysis is therefore meaningful only at λ=9 and 15, where the shell is well-defined. There, the first-shell occupancy confirms the two-stage picture of $g_{1}$: it is high below the drift threshold and collapses once the drift regime is entered. For λ=15, the number of coordinating sulfonic groups rises from 1.16±0.03 at E=0 to 1.65±0.13 near the drift-onset field (E=3.5 V ${\mathrm{nm}}^{-1}$) and falls to 1.10±0.16 at E=4.0 V ${\mathrm{nm}}^{-1}$; for λ=9, it remains at ≈2.8–3.2 up to the drift onset (3.24±0.15 at E=0; 2.80±0.22 and 3.03±0.46 at E=1.4 and 1.5 V ${\mathrm{nm}}^{-1}$) and drops to 1.89±0.37 at E=2.0 V ${\mathrm{nm}}^{-1}$, beyond the onset, where *D* has already jumped by two orders of magnitude. The nonmonotonic $g_{1}$ signature therefore reflects a genuine change in first-shell occupancy, not a normalization artifact, and the drift onset coincides with the loss of hydronium–sulfonate coordination. The S–S and S–W coordination numbers show no counterpart of the S–H_3_O^+^ collapse, varying by ≲16% over the full field range: the S–S number decreases monotonically with hydration (2.72±0.09, 1.87±0.07, and 1.77±0.04 for λ=3, 9, and 15 at E=0), reflecting the dilution of the sulfonate network, whereas the number of water molecules in the first sulfur shell is largest at high hydration (≈7.0, 6.6, and 11.3 for λ=3, 9, and 15; the λ=3 value is inflated by its wider first shell, $r_{c}\approx 0.87$ nm versus 0.66 nm at λ=9), reflecting the growing hydration of the sulfur environment, consistent with Figure 9.

## 4. Conclusions

At zero field, the static dielectric constant of hydrated Nafion is set by collective dipolar correlations: the aqueous-subsystem ϵ grows with hydration (from ≈2.9 at λ=3 to ≈17.2 at λ=15), while the single-molecule dipole remains essentially constant, so the enhancement is cooperative in origin and tracks the percolation of the aqueous domains. Under an external field, the dipole-fluctuation formula no longer defines a dielectric constant; the meaningful response is the polarization itself, which we follow through the longitudinal dipole $\langle M_{z}\rangle$ and the alignment fraction $|M_{z}\left|/|M\right|$. The orientation of the collective water dipole locks onto the field already at low fields for every hydration level; the field then progressively saturates the magnitude of the water polarization, reorganizes the first H_3_O^+^–SO_3_(H) coordination shell, and drives a crossover from diffusive to drift-dominated proton transport. The central result is the coupling among these responses. The saturation field of the water polarization, the first-shell occupancy, and the transport threshold all move to higher fields as hydration increases, and they move together. We attribute this concerted shift to electrostatic screening: the added water weakens the sulfonate–ion interaction, and, in doing so, it sets at once the equilibrium dielectric response and the field required for directed hydronium transport. Because all systems share a fixed simulation cell, the mass density increases with hydration, and the absolute diffusion coefficients (in particular, their decrease with λ) reflect this controlled-volume protocol rather than the behavior of a freely swelling membrane. Our conclusions concern the coupling between the polarization, structural, and transport responses and the concerted shift of their characteristic field with hydration, which are robust to this choice; they are not intended as predictions of absolute mobilities or of the hydration dependence of conductivity at each equilibrium density. Viewed this way, the dielectric, structural, and transport signatures form a single picture of the field response of perfluorosulfonated membranes, one in which hydration acts as a single knob that tunes both the polarization and the onset of proton conduction under bias.

From a practical standpoint, the coupled onset fields identified here are relevant wherever local electric fields in an operating membrane can approach or exceed the values probed in this work, such as near catalyst-layer/membrane interfaces or across thin regions of locally low hydration. In particular, the low-hydration membrane (λ=3) crosses from diffusive to drift-dominated hydronium transport at a markedly lower field (E≈0.7 V ${\mathrm{nm}}^{-1}$) than the well-hydrated membrane (λ=15, E≈3.7 V ${\mathrm{nm}}^{-1}$); a locally dehydrated region of the membrane is therefore disproportionately susceptible to field-driven, non-diffusive transport. This suggests that maintaining uniform hydration, rather than only a high average hydration, is important for keeping proton transport in the diffusive regime under the local fields present in an operating fuel cell, a design consideration that follows directly from the coupling reported here.

A natural extension of this work would be to repeat the present λ×E scan under constant-pressure (NPT) conditions, allowing each hydration level to swell to its own equilibrium volume; this would test directly whether the coupling and the field thresholds reported here persist, and how they shift, once the fixed-volume constraint used in this study is lifted.

## Figures and Tables

**Figure 1:**
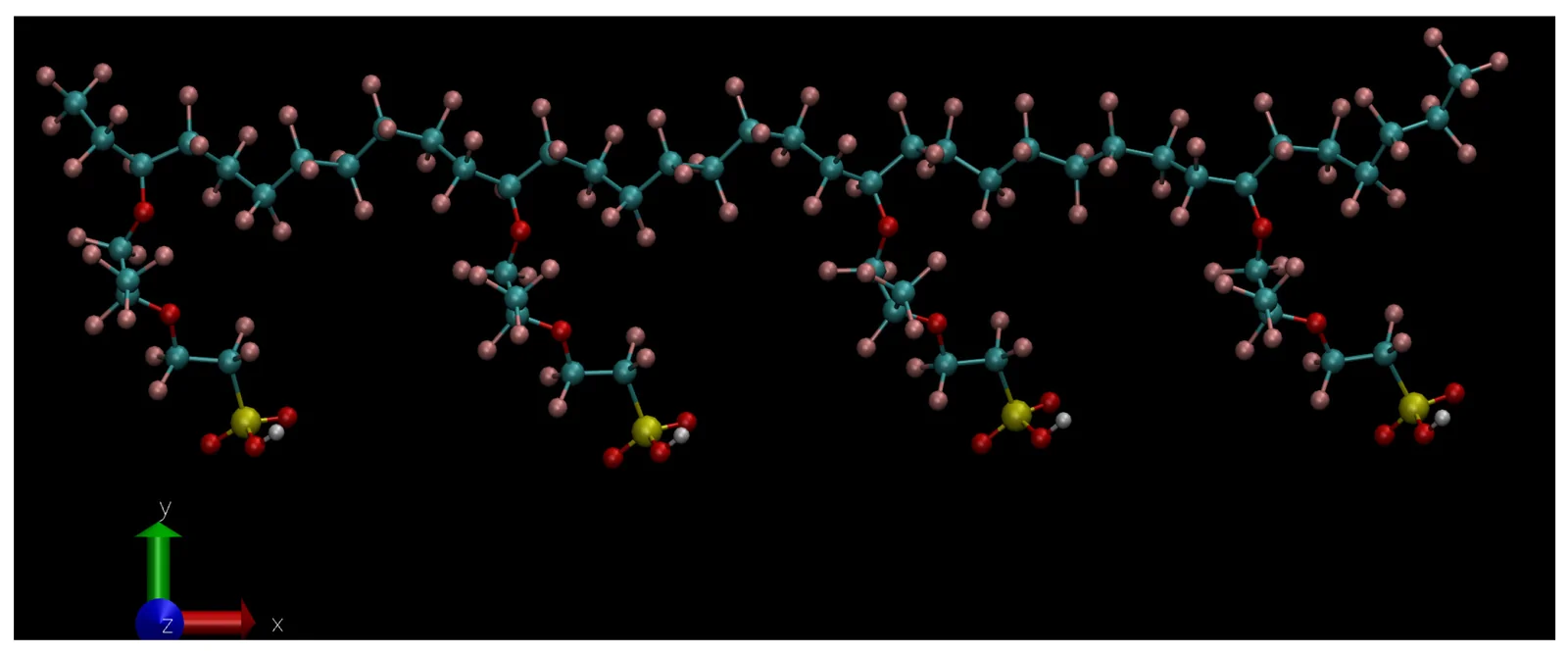
Snapshot of molecular model of single Nafion polymer.

**Figure 2:**
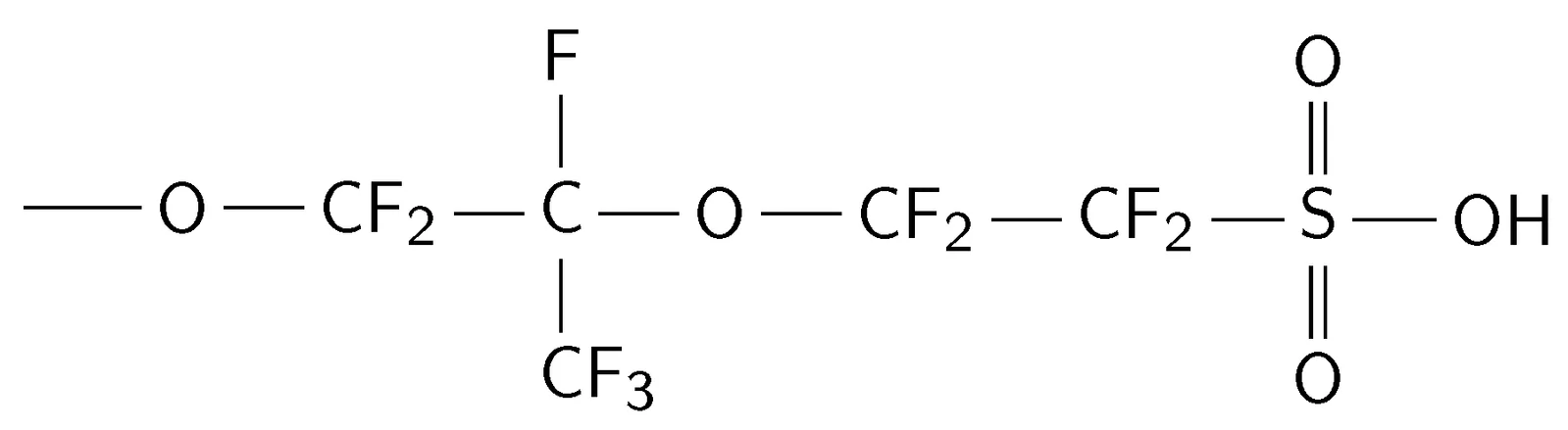
Chemical structure of the perfluoroether side chain terminated by a sulfonic acid group ($\text{-}{\mathrm{SO}}_{3}H$). Nafion monomer used in the simulations consists of a backbone formed by perfluorinated units (CF3-CF2-CF-(CF2)8-CF-(CF2)8-CF-(CF2)8-CF-(CF2)5-CF3) with 4 side chains attached to the 4 CF units.

**Figure 3:**
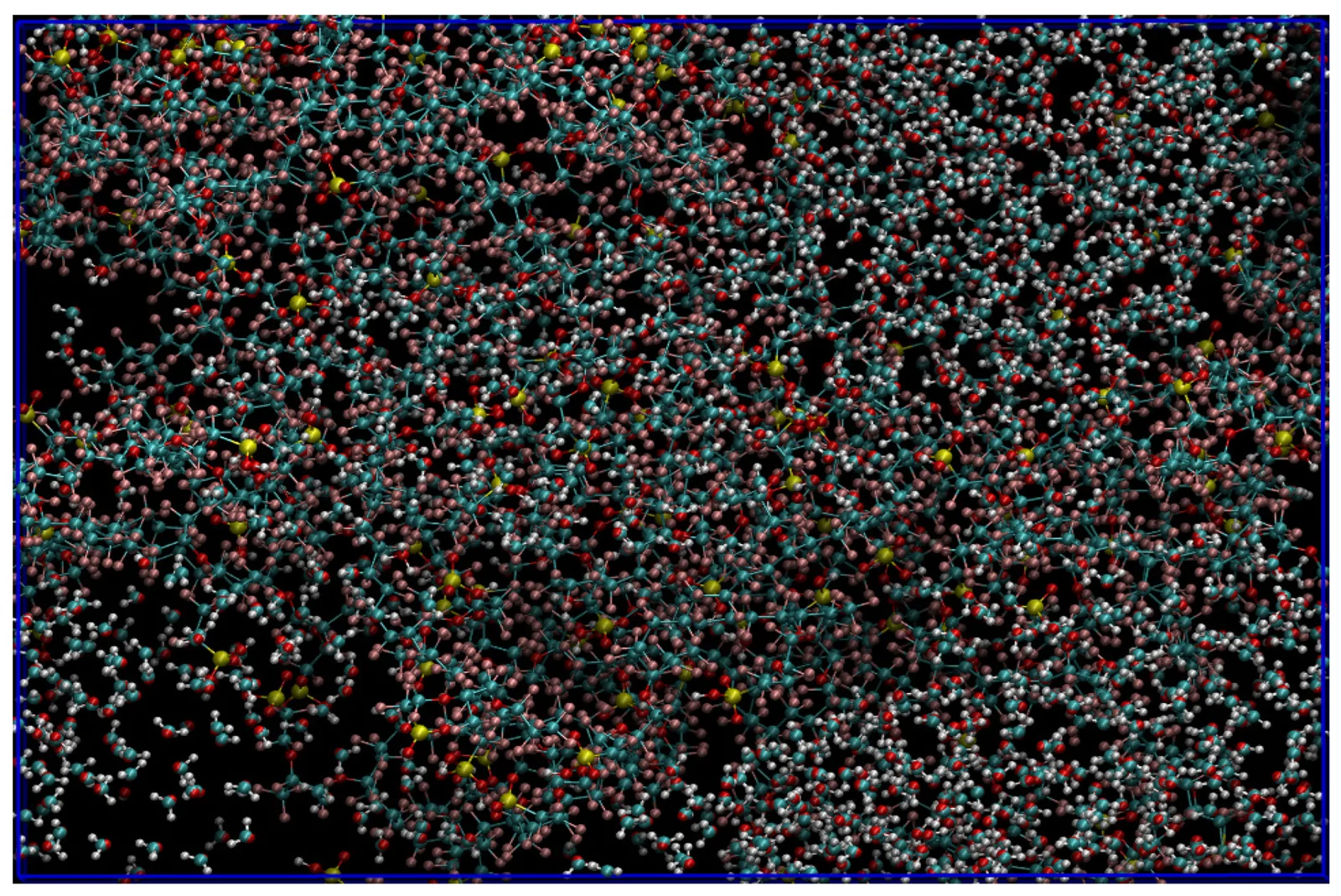
Snapshot of the system with λ=9.

**Figure 4:**
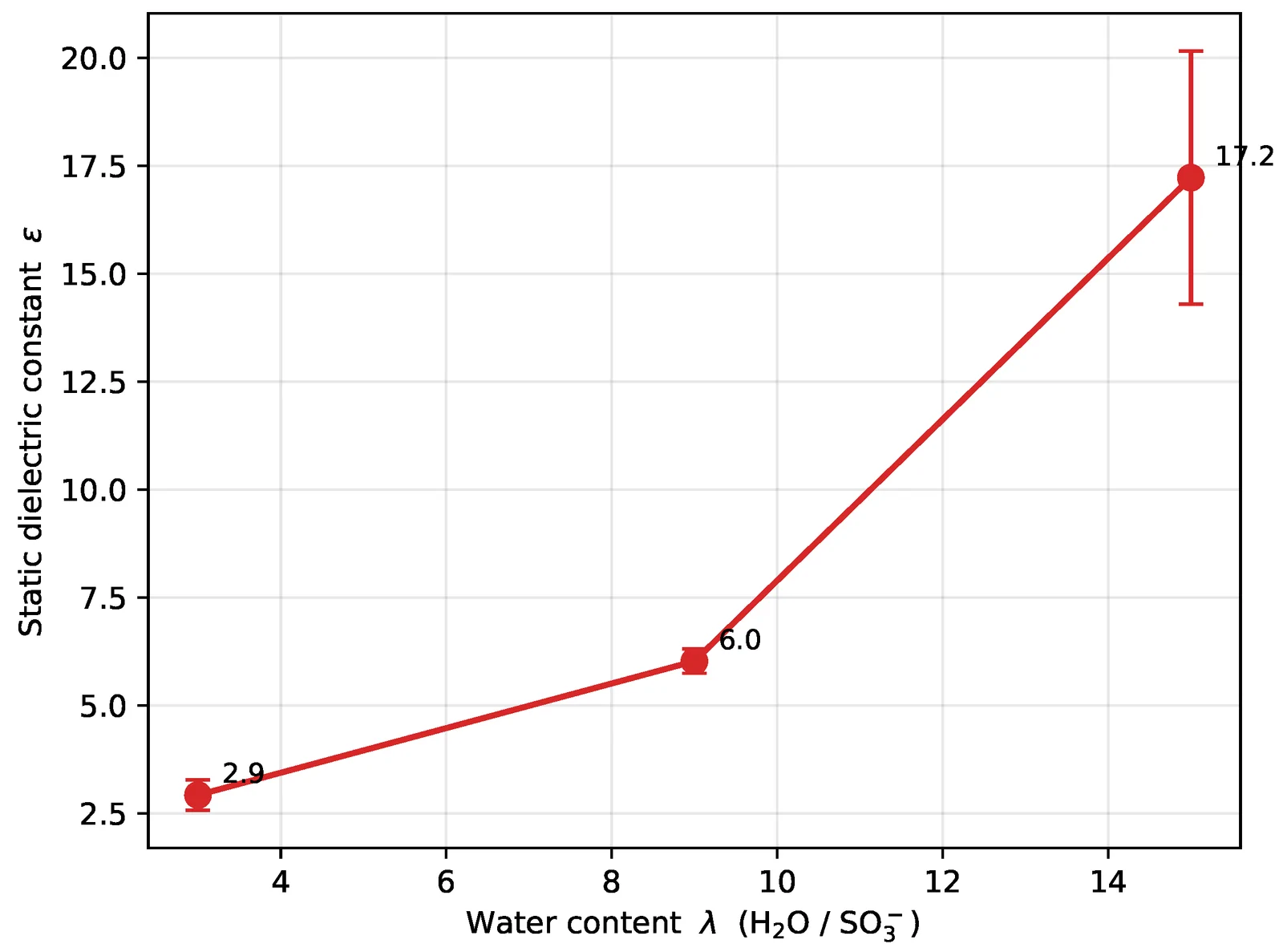
Static dielectric constant ϵ of the aqueous subsystem as a function of hydration level λ at zero field, obtained from the Kirkwood–Fröhlich dipole fluctuation formula applied to the water molecules only. This is the only field condition under which the fluctuation formula defines an equilibrium dielectric constant; ϵ increases from ≈2.9 at λ=3 to ≈17.2 at λ=15, driven by the collective dipolar correlations that develop as the aqueous domains percolate. Error bars are the standard deviation across four 2 ns blocks; the scatter is largest at λ=15, where the slowly relaxing collective dipole is hardest to converge within the fixed sampling window.

**Figure 5:**
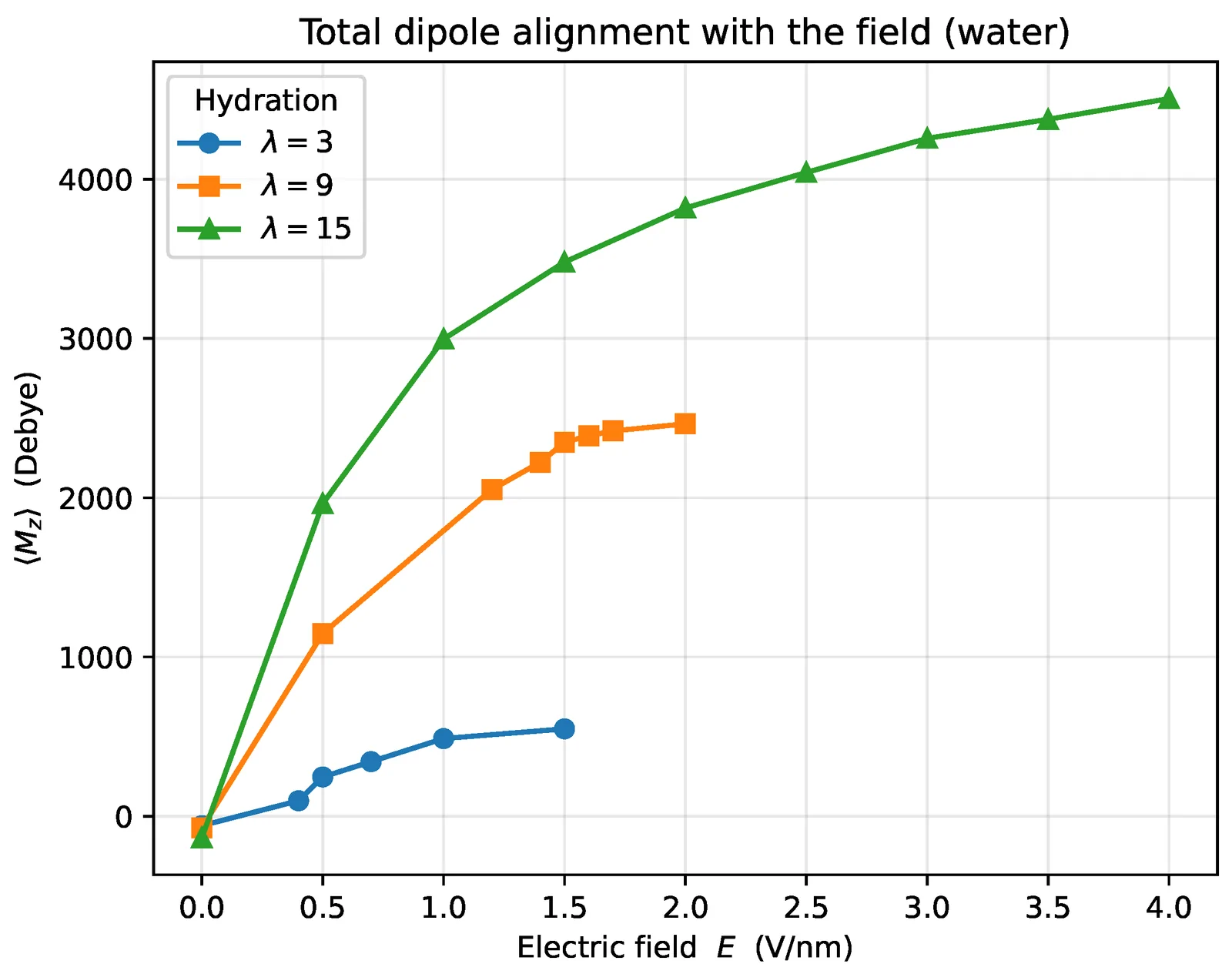
Longitudinal total dipole $\langle M_{z}\rangle$ of the aqueous subsystem vs. applied field *E* for each λ. The rise is steep at low fields, and the approach to saturation is gradual; both the saturated magnitude and the field needed to reach it grow with hydration (E≈1.5, 2, and 3–4 V ${\mathrm{nm}}^{-1}$ for λ=3, 9, and 15, respectively).

**Figure 6:**
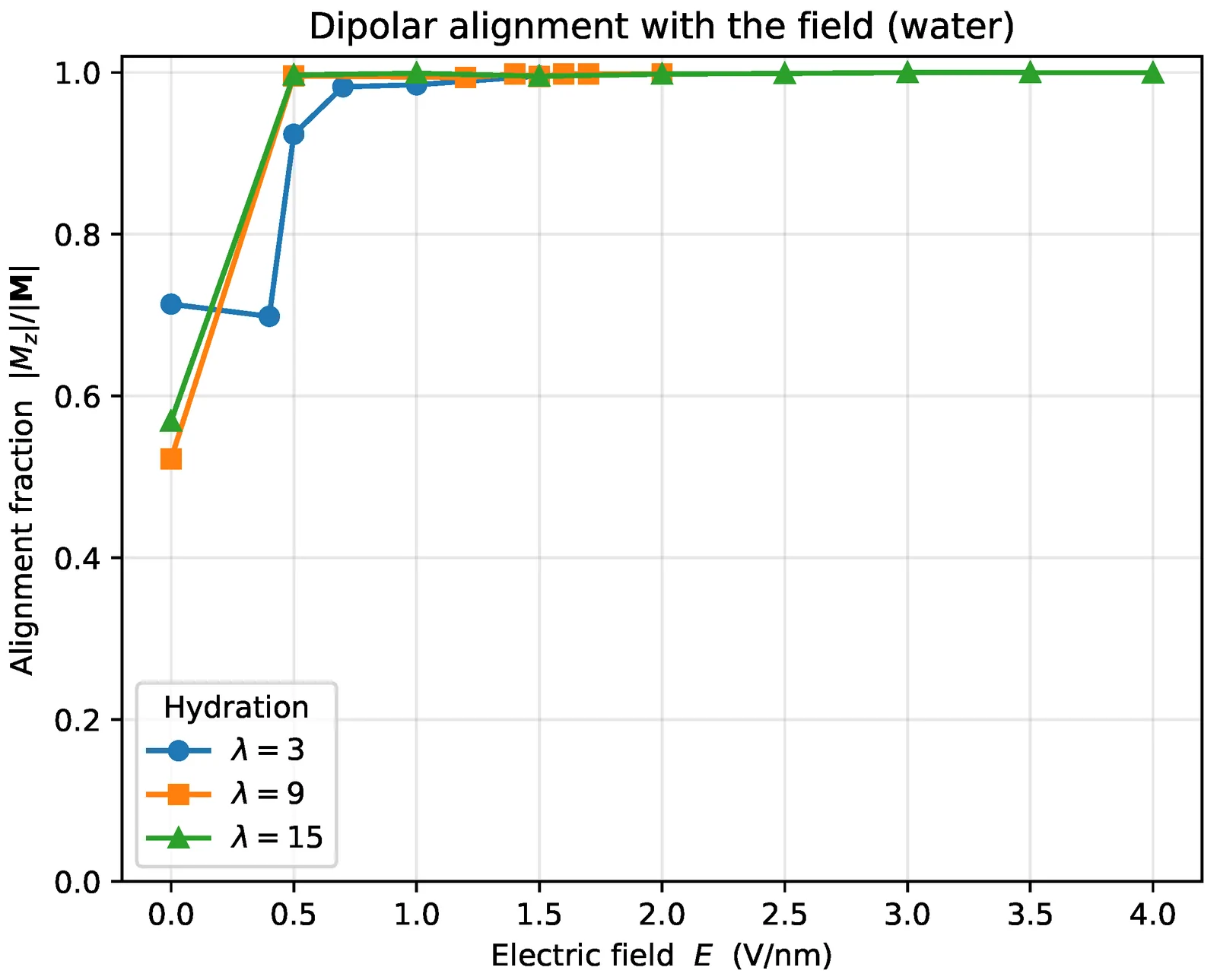
Alignment fraction $|M_{z}\left|/|M\right|$ of the aqueous subsystem (computed per frame and averaged) vs. applied field *E* for each λ. It rises from the near-isotropic zero-field value (≈0.5–0.7) to ≳0.99 already at E=0.5 V ${\mathrm{nm}}^{-1}$ for λ=9 and 15, and by E≈0.7 V ${\mathrm{nm}}^{-1}$ for λ=3: the orientation of the collective water dipole locks onto the field at fields well below those at which its magnitude saturates (cf. Figure 5).

**Figure 7:**
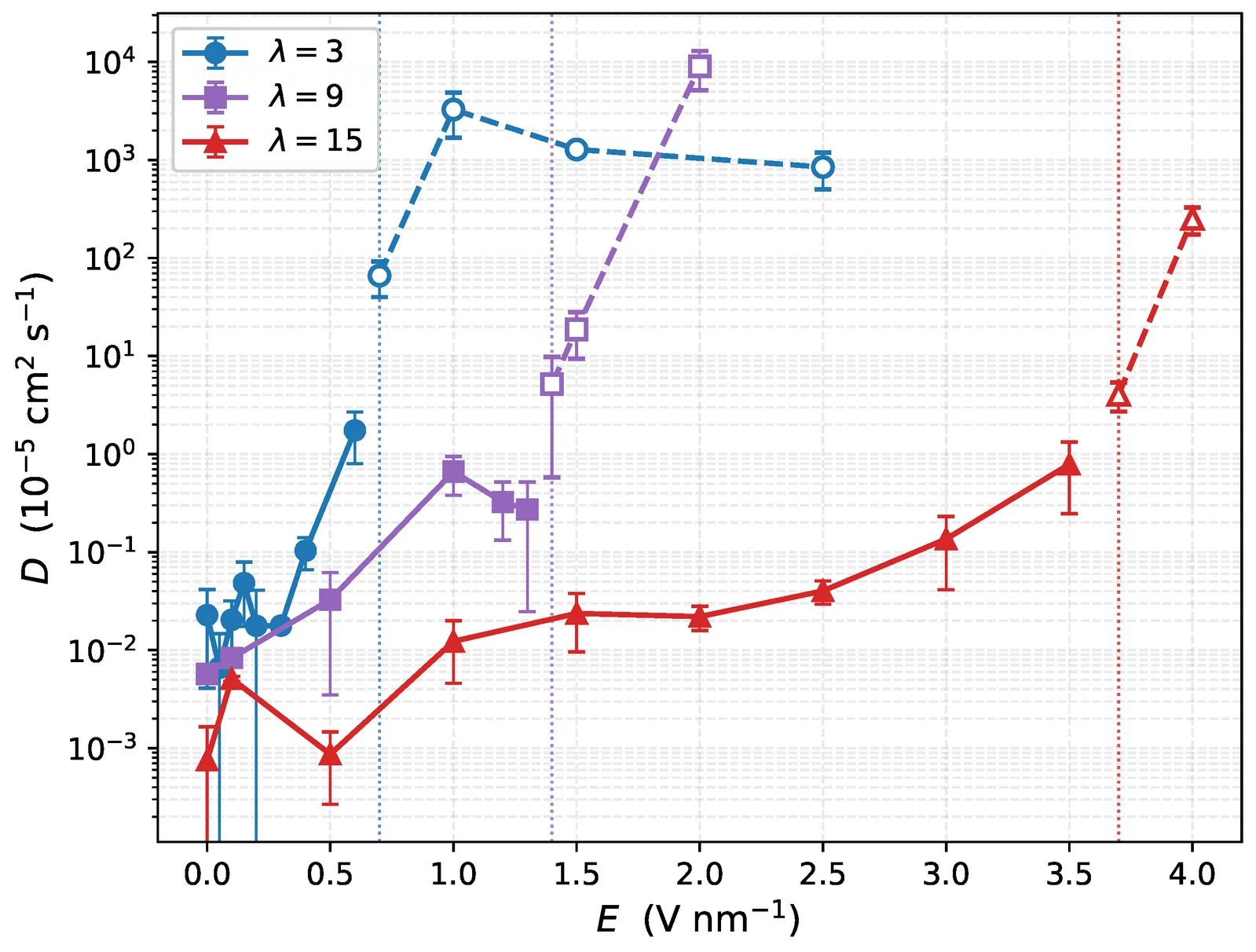
Diffusion coefficient *D* of H_3_O^+^ as a function of the applied electric field *E* for λ=3, 9 and 15 (logarithmic vertical scale). Filled markers with solid lines denote the diffusive regime used in the quantitative analysis; open markers with dashed lines denote the drift-dominated regime, where the apparent *D* jumps by several orders of magnitude. Vertical dotted lines mark the onset of field-driven drift, which shifts to higher fields as λ increases (≈0.7, 1.4 and $3.7\;V\;{\mathrm{nm}}^{-1}$ for λ=3, 9 and 15). The drift-regime points are shown to demonstrate the onset but are excluded from the diffusive analysis.

**Figure 8:**
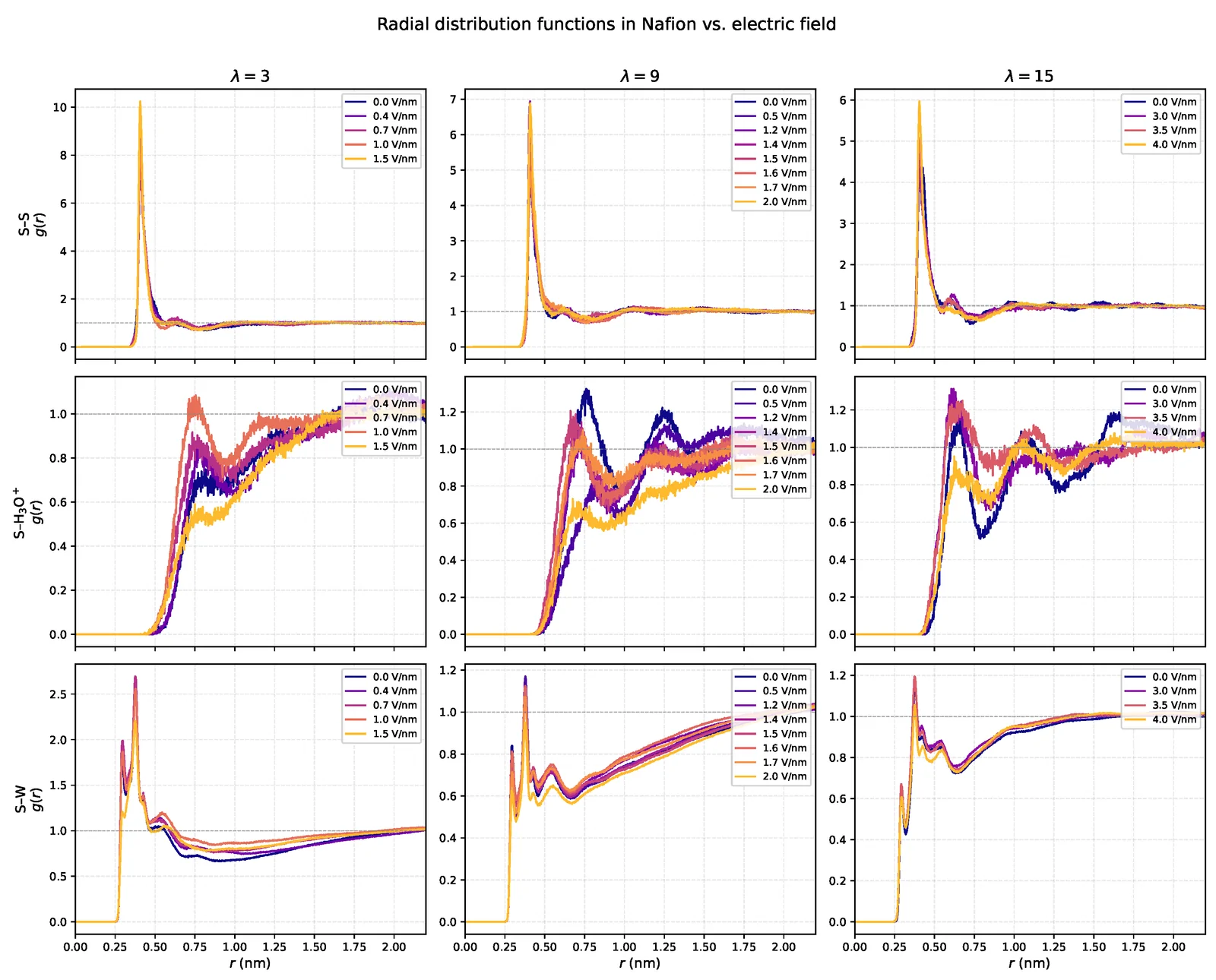
Radial distribution functions g(r) for S–S (**top row**), S–H_3_O^+^ (**middle row**), and S–W (**bottom row**) pairs for λ=3, 9, and 15 (columns) at several applied electric fields (color scale from dark to light: low to high *E*). The S–S and S–W distributions are essentially field-independent below the drift onset for all hydration levels, indicating that the polymer backbone and the primary hydration shell of the sulfonate groups are not significantly restructured by the field. The S–H_3_O^+^ distribution, by contrast, shows a systematic nonmonotonic evolution with *E*, discussed quantitatively in Figure 9.

**Figure 9:**
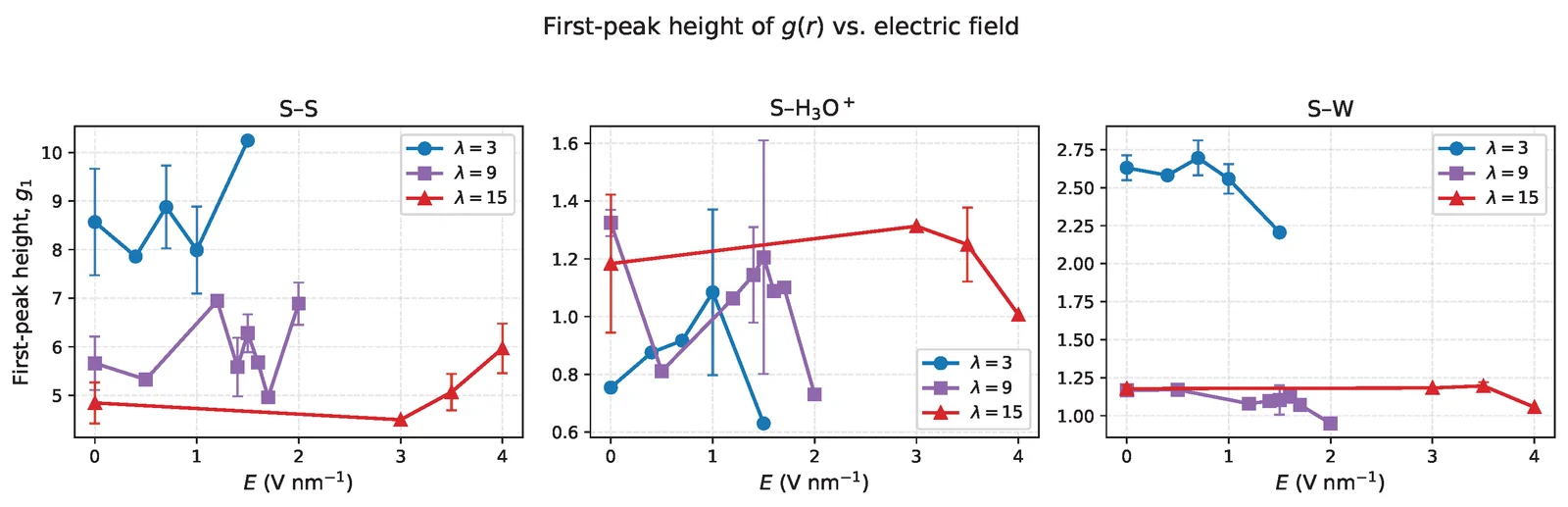
Height of the first peak $g_{1}$ of the radial distribution functions as a function of applied field *E* for S–S (**left**), S–H_3_O^+^ (**center**), and S–W (**right**) for λ=3, 9, and 15. While the S–S and S–W peak heights show no systematic field dependence below the drift onset, the S–H_3_O^+^ peak height is nonmonotonic in the field for all λ: it rises (for λ=9 after an initial low-field dip) toward a maximum near the drift-onset field and then drops once the drift regime is entered (see Figure 7). This two-stage evolution identifies the crossover from localized coordination to delocalized transport as the microscopic origin of the coupling between structure and transport. Error bars, shown where computed, are the standard deviation of $g_{1}$ over four 2 ns blocks; they are largest for S–H_3_O^+^, reflecting the limited statistics of the dilute hydronium population.

**Table 1:** Molecular dynamics simulation parameters, common to all hydration levels and field strengths.

Quantity	Value
MD engine	GROMACS 2024.5
Integrator	leap-frog
Time step	0.5 fs
Equilibration	$5\times 10^{6}$ steps (2.5 ns)
Production	$2\times 10^{7}$ steps (10 ns)
Ensemble	NVT (fixed volume)
Thermostat	Nosé–Hoover ($\tau_{T}=0.05$ ps)
Temperature	300 K
Box (fixed)	4.80×4.50×6.80 nm3
Cutoff scheme	Verlet
$r_{\mathrm{Coulomb}}=r_{\mathrm{vdW}}$	1.0 nm
Long-range electrostatics	PME
Dispersion correction	energy + pressure
Constraints	LINCS (order 8, 2 iter.)
Water model	TIP4P/Ice

**Table 2:** Diffusion coefficients *D* of hydronium ions as a function of hydration level λ and applied electric field *E*, from linear fits to the MSD curves over 2000–6000 ps. For each λ, the upper block corresponds to the diffusive regime used in the analysis; the last entries (marked ^†^) lie beyond the drift onset, where the apparent *D* is dominated by field-driven drift, and are listed for completeness only.

λ	*E* (${\mathrm{V nm}}^{-1}$)	*D* ($10^{-5}$ cm2 s−1)
3	0.0	0.0228
3	0.1	0.0204
3	0.2	0.0177
3	0.3	0.0178
3	0.4	0.103
3	0.6	1.74
3	$0.7^{\;†}$	66.19
9	0.0	0.0057
9	0.5	0.0327
9	1.0	0.6624
9	1.2	0.325
9	1.3	0.2724
9	$1.4^{\;†}$	5.17
9	$1.6^{\;†}$	80.69
15	0.0	0.000075
15	0.1	0.005106
15	1.0	0.0123
15	1.5	0.023
15	2.0	0.022
15	2.5	0.0402
15	3.0	0.1361
15	3.5	0.7874
15	$3.7^{\;†}$	4.0412
15	$4.0^{\;†}$	250.29

## Data Availability

The GROMACS input files (topologies and run parameters) and the raw simulation outputs underlying the figures and tables (λ = 3, 9, and 15, the hydration levels discussed in this work) are openly available in Zenodo at https://doi.org/10.5281/zenodo.22309731. The full production trajectories, which amount to several hundred gigabytes, are not included in that deposit for storage-size reasons; they are archived at the Laboratorio Nacional de Supercómputo del Sureste de México and are available from the corresponding author upon reasonable request.

## Abbreviations

The following abbreviations are used in this manuscript:

PEMFC	Proton exchange membrane fuel cell
PFSA	Perfluorosulfonic acid
PTFE	Polytetrafluoroethylene
MD	Molecular dynamics
DFT	Density functional theory
ATB	Automated Topology Builder
EW	Equivalent weight
NVT	Canonical ensemble (constant number, volume, temperature)
PME	Particle Mesh Ewald
MSD	Mean squared displacement
RDF	Radial distribution function
MS-EVB	Multistate empirical valence bond
LINCS	LINear Constraint Solver
